# Microbubble Enhanced Delivery of Vitamin C for Treatment of Colorectal Cancer

**DOI:** 10.1021/acsomega.4c06779

**Published:** 2024-10-30

**Authors:** Joseph Fox, Damien V. B. Batchelor, Patricia Louise Coletta, Elizabeth M.A. Valleley, Stephen D. Evans

**Affiliations:** † Molecular and Nanoscale Physics Group, School of Physics and Astronomy, University of Leeds, Leeds LS2 9JT, U.K.; ‡ Leeds Institute of Medical Research, St James’s University Hospital, Wellcome Trust Brenner Building, Leeds LS9 7TF, U.K.

## Abstract

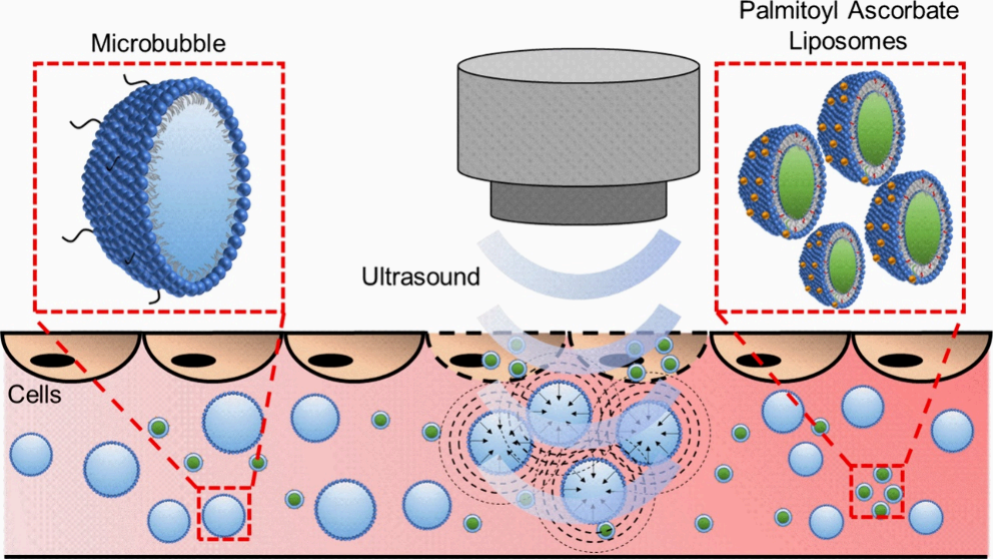

During chemotherapy treatment for cancer, often only a fraction of the administered dose reaches the tumor site, with the remaining drug spreading throughout the body, producing unwanted side-effects and restricting how much drug can be safely administered. A potential solution to reduce this problem is the use of microbubbles. The interaction between microbubbles and ultrasound generates pores in the tumor cells, permitting enhanced drug uptake. This study investigates the delivery of the ascorbic acid derivative, palmitoyl ascorbate, to KRAS-mutated colorectal cancer cells in vitro. Ultrasound-triggered microbubbles enhanced the efficacy of liposomal palmitoyl ascorbate treatments by 1.7- and 2.2-fold in LS174T and HCT116 CRC cell lines, respectively. This enhancement was achieved without increasing the drug dosage, and the therapeutic effect was shown to be localized to the area that received the ultrasound pulse, aiding in the reduction of off-site toxicity.

## 1 Introduction

Colorectal cancer (CRC) is the third most common malignancy and the second leading cause of cancer death globally,^1^ with 1.8 million new cases diagnosed in 2018.^2^ In the case of advanced or metastatic disease, the chemotherapy regimens typically followed use mixtures of drugs (e.g., FOLFOX or FOLFIRI^3^). However, such treatments can be systemically damaging, with side effects including an increased infection risk, peripheral neuropathy, nausea, diarrhea, alopecia^4^ and the loss of fertility in both men and women.^5^

High doses of vitamin C (ascorbic acid, AA) have shown selective anti-cancer effects^6^ and have improved the efficacy of various chemotherapy drugs.^7−9^ KRAS and BRAF mutations occur in 40%^10^ and 10%^11^ of CRC cases, respectively, and are linked to chemoresistance and adverse clinical outcomes.^12^ KRAS and BRAF affect cells by altering the cellular glucose metabolism by upregulating the glucose transporter 1 (GLUT-1).^13^ Vitamin C is particularly cytotoxic toward KRAS and BRAF mutated CRCs due to an increased uptake of the oxidized form of AA (dehydroascorbic acid) via GLUT-1, which is abundant in the mutated cells.^14^ In vivo applications of vitamin C have been limited by the high plasma concentrations needed to achieve efficacy due to strict physiologic regulation and the chemical instability of ascorbate in the bloodstream.^15^ High levels (0.5–5 mM) of vitamin C can be obtained by intravenous administration, though this carries the risk of promoting thrombosis.^16^ Hence, providing a targeted delivery of vitamin C has significant promise.

This work explores microbubble (MB) enhanced delivery of liposomal vitamin C to CRC cell lines. MBs are typically 1–10 μm sized particles formed with a gas core and typically a lipid shell.^17^ MBs are theranostic agents in that they can be visualized upon US exposure and hence have a diagnostic property coupled with their therapeutic capabilities.^18^ Upon exposure to the US, MBs undergo volumetric oscillations at the same frequency as the US excitation.^19^ At low acoustic pressures, the amplitude of the oscillations is small, and the MBs undergo stable cavitation.^20^ These oscillations cause microstreaming in the surrounding fluid, which can cause shear stress on nearby cell membranes, leading to tension and stretching of the membrane walls or junctions between cells.^21^ At high amplitudes, the US causes the MBs to undergo large size fluctuations, potentially leading to their destruction through inertial cavitation.^22^ Inertial cavitation can create radial shock waves or jets,^23^ leading to cell poration. A 1–10 μm MB possesses a resonance frequency of 10–1 MHz,^24^ aligning well with the range of frequencies used in medical US imaging.^25^ Both stable cavitation^21^ and inertial cavitation^26^ processes can generate “sonoporation”,^27^ the formation of pores in cell membranes, which leads to the enhanced permeability of the cells and which, combined with therapeutic agents, can enhance drug uptake.

Due to the instability of aqueous vitamin C in the bloodstream, research focus has shifted toward using palmitoyl ascorbate (PA).^15,28−33^ PA is a commercially available, hydrophobized vitamin C derivative that can be incorporated into the liposomal bilayer,^15^ with the hydrophobic palmitoyl chain sitting within the acyl chains of the lipids^31^ while the hydrophilic ascorbate part of the molecule is situated in the outer liposomal shell. D’Souza et al.^15^ demonstrated that palmitoyl ascorbate liposomes (PAL) (1.6–7.5 mM) were toxic to human ovarian, breast, and kidney cancer cell lines. Paclitaxel incorporated into PAL showed significantly increased cytotoxicity over the equivalent concentration of paclitaxel incorporated into non-PA liposomes when tested against BT20 (breast), MCF-7 (breast), RAG (mouse renal), and A2780 (ovarian) cancer cells in vitro^15^ and a 4T1 mouse breast tumor model in vivo*.*^33^ Sawant et al.^30^ used 4T1 murine mammary carcinoma cells to study in vitro cytotoxicity of PAL (1.875 and 3.75 mM) and free AA combinations. In all cases, the PAL treatments showed increased cell death over AA alone. In vivo studies demonstrated that PAL generated a more significant cytotoxic effect than AA, even when given at a lower, less frequent dose. Yang et al.^28^ showed that loading hydrophobic doxorubicin into PAL gave an enhanced in vitro cytotoxic effect on human MCF-7 (breast), HepG2 (liver), and A549 (lung) cells in comparison to either doxorubicin or PA liposomes alone. Li et al.^29^ investigated liposomes containing PA and docetaxel and found that when compared to docetaxel liposomes, the combination of PA and docetaxel showed enhanced tumor cell killing in all three cell lines studied - HepG2 (liver), MCF-7 (breast), and PC-3 (prostate).^29^

This work used LS174T and HCT116 CRC cell lines, both of which possess KRAS mutations,^34^ to demonstrate that the therapeutic effect of PAL can be enhanced by delivery via the MB and US platform. It is shown that multiple exposures to MBs and US amplify PAL’s localized therapeutic efficacy without raising the drug dose or exposure time.

## 2 Methods

### 2.1 Materials

High glucose Dulbecco’s modified eagle medium (DMEM, 41965039), Glutamax (35050038), and Dulbecco’s phosphate-buffered saline (DPBS, 14190–094) were purchased from Thermo Fisher. Fetal bovine serum (FBS, F7524), l-Ascorbic acid (AA, A92902), cholesterol (C8667), and 6-O-Palmitoyl-l-ascorbic acid (PA, 76183) were purchased from Sigma. 1,2-Dipalmitoyl-*sn*-glycero-3-phosphocholine (DPPC, 850355p) and 1,2-distearoyl-*sn*-glycero-3-phosphoethanolamine-N-[methoxy(polyethylene glycol)-2000] (DSPE-PEG2000, 880120P) were purchased from Avanti Polar Lipids. 96-well plates (655–180) were purchased from Greiner Bio-One. Phosphate buffered saline (PBS, 003002) was purchased from Invitrogen. Glycerol (BP229) was purchased from Fisher Scientific. The 20 mm gel standoff pad (04–02) was purchased from AquaFlex. US coupling gel (UGEL1000) was purchased from Ana Wiz. Microfluidic devices (μ-Slide VI 0.4 ibiTreat, 80606) were purchased from Ibidi. CellTiter 96 Aqueous One Solution Cell Proliferation Assay (3-(4,5-dimethylthiazol-2-yl)-5-(3-carboxymethoxyphenyl)-2-(4-sulfophenyl)-2H-tetrazolium, inner salt) MTS reagent (G358C) was purchased from Promega and used to assess cell viability. Calcein-AM and Ethidium Homodimer III (30002) were purchased from Biotium.

### 2.2 Cell Culture

LS174T human Caucasian colon adenocarcinoma (ECACC 87060401) and HCT116 human colon carcinoma (ECACC 91091005) cells were initially obtained from the European Collection of Authenticated Cell Cultures (ECACC, UK). Cells were grown and maintained in high glucose DMEM supplemented with 10% FBS and 1% Glutamax in an incubator at 37 °C with 5% CO_2_.

### 2.3 LS174T Cell Response to Free AA

LS174T cells were seeded in a 96-well plate at a seeding density of 4000 cells per well. 24 h after seeding, cells were washed with 100 μL DMEM. A 40 mg/mL AA stock solution in DPBS was made and filtered (0.2 μm) under sterile conditions. Cells were incubated with AA at a range of concentrations for 1 h, at 100 μL final volume. Carrier controls were made containing the same volume concentration of DPBS and DMEM as used in the treatment solution to confirm no cytotoxicity was caused by the vehicle. All treatments and the carrier control contained 8.8% (v/v) DPBS in the final formulation, which showed no significant effect on cell viability when compared to DMEM alone. Post-exposure to AA, cells were washed twice with DMEM and incubated for 24 h before adding 20 μL MTS reagent to each well. Cells were returned to the incubator for 4 h before the absorbance at 490 nm was recorded using a SpectraMax m2e well plate reader (WPR). DMEM and MTS reagent combined in empty wells were used to measure the MTS background signal. Three biological repeats were conducted, each performed from a different culture flask; each contained 3 intra-experimental repeats with new drug solutions. Cell viability was calculated using eq 1, in which *A*_Sample_, is the average absorbance of treated cells, *A*_BG_, is the average background absorbance of wells containing MTS and DMEM but no cells, and *A*_Carrier_ is the average absorbance of cells treated with the carrier control.
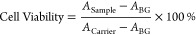
1

### 2.4 PAL Preparation and Characterization

PAL were prepared using DPPC, cholesterol, and PA in the molar ratio 49:21:30, with molar ratios chosen to align with the works of Sawant at al.^30^ and D’Souza et al.^15^ Organic solvent was removed by drying under nitrogen until visibly dry (1–2 h), then placing in a vacuum desiccator overnight. The dried lipid film was resuspended in 1 mL PBS and put on a heater stirrer at 55 °C, 650 rpm for 30 min, giving a final lipid concentration of 10 mg/mL. PAL were extruded sequentially through 400 and 200 nm membranes and then filtered under sterile conditions (0.2 μm) before use on cells. Blank liposomes (BL) were prepared the same way, with the omission of PA. Liposome size and concentration were determined using a NanoSight NS300 (Malvern Panalytical). High-performance liquid chromatography (HPLC) and ultraviolet–visible-near-infrared (UV–vis-NIR) spectroscopy were used to determine the PA concentration present in PAL. HPLC analysis was performed using an Agilent 1290 Infinity II HPLC system (Agilent, USA) with a diode array detector (DAD). Chromatographic separations were performed using an Agilent InfinityLab Poroshell 120 EC-C18 (2.1 × 5 mm, 1.9 μm) at a column temperature of 40 °C. The mobile phase used was 0.1% H_3_PO_4_ in water (10%) and an acetonitrile/methanol–water mixture (40:55:5) with 0.1% H_3_PO_4_ (90%) over 5 min at a flow rate of 0.5 mL/min. The DAD recorded the chromatogram at 210 and 254 nm wavelengths, and the injection volume used was 1 μL. A calibration curve between PAL samples with known absorbance peaks was recorded on a UV–vis-NIR spectrophotometer (Cary 5000, Agilent USA), and corresponding HPLC measurements were generated. Hence, the concentration of PAL could be calculated from UV–vis spectra.

### 2.5 LS174T Cell Response to PAL

LS174T cells were exposed to PAL following the same treatment schedule detailed in Section 2.3. PAL and BL were prepared using the methods outlined in Section 2.4, filtered under sterile conditions (0.2 μm), then concentrated 10-fold by centrifugation at 17,000*g* for 30 min before removing 90% of the supernatant and vortex mixing the remaining pellet. The concentration of PBS was held constant across all conditions tested, and a PBS and DMEM carrier control was tested to discern any cytotoxicity from exposure to the PBS vehicle used for PAL and BL delivery. Three biological repeats were conducted, each from a different culture flask, using separately made, fresh-prepared liposomal solutions. Each biological repeat contained 3 intra-experimental repeats. Statistical analysis was performed using GraphPad Prism 9 (GraphPad Software, Inc., California, USA). One-way analysis of variance (ANOVA) tests (Tukey’s multiple comparisons) were used to assess statistical significance, with *p* < 0.05 considered statistically significant and significance represented in plots by *p < 0.05, **p < 0.01, ***p < 0.001 and ****p < 0.0001.

### 2.6 Microfluidic MB Production and Characterization

MBs were prepared using DPPC and DSPE-PEG2000 in the molar ratio 95:5. Organic solvent was removed by drying under nitrogen until visibly dry (1–2 h), then placing in a vacuum desiccator overnight. The dried lipid film was resuspended in 2 mL PBS containing 1% (v/v) glycerol and put on a heater stirrer (Heidolph, MR Hei-Tec) at 55 °C, 650 rpm for 20 min, giving a 2 mg/mL final lipid concentration. The lipid solution was tip sonicated (20 kHz, 150 W, Sonifier 250, Branson, USA) for 40 min at 4 °C and the solution was centrifuged at 17,000*g* for 5 min to remove any titanium deposited during tip sonication. Lipid solution was then combined with C_4_F_10_ gas in a microfluidic device for MB production as described previously^35,36^ using a liquid flow rate of 100 μL/min and a gas pressure of 1000 mbar. MBs were sized and counted using bright-field microscopy (Nikon 90i, Nikon, Japan) and a custom MATLAB script.^37^

### 2.7 On-Chip PAL + MB + US Treatments

On-chip studies were conducted using a variation of the methods of Batchelor et al.^38^ 30 μL of LS174T or HCT116 cell suspension was seeded into the channels of an Ibidi μ-Slide VI microfluidic device at a concentration of 7 × 10^5^ cells/mL. The device was inverted and incubated for 3 h to allow cells to adhere to the top face of the microfluidic channels. After 3 h, the device was righted, and 60 μL DMEM was slowly added to both reservoirs of each channel simultaneously. After 24 h, cell channels were washed with 3 × 100 μL DMEM, then 3 × 50 μL of treatment formulation was added. The triplicate addition and removal of liquid was conducted to ensure the microfluidic channel contained the treatment formulation. PAL and BL were prepared using the methods outlined in Section 2.4, filtered under sterile conditions (0.2 μm), then concentrated 10-fold by centrifugation at 17,000*g* for 45 min before removing 90% of the supernatant and vortex mixing the remaining pellet. MBs were prepared using the methods outlined in Section 2.6, then UV sterilized for 30 min. MBs introduced to microfluidic channels were allowed to rise for 10 min before US exposure, with rise time informed by models of the Hadamard-Rybczynski equation.^39^ PAL and MBs were diluted in DMEM to give a final concentration of 5 mM and 1 × 10^8^ MBs/mL, respectively. In the multi-exposure treatments, 3 × 50 μL of new formulation was added to the channel and given 10 min rise time before US exposure. 1× refers to one formulation addition and one ultrasound exposure, 3× refers to three formulation renewals and three ultrasound exposures and 5× refers to five formulation renewals and five ultrasound exposures. A 2 h total drug exposure time was held constant across the channels, starting from the application of the first formulation. In all controls using MBs, MBs are at the same final concentration as the treatment formulation (1 × 10^8^ MBs/mL). In all controls using PAL, PAL is at the same final concentration as the treatment formulation (5 mM). After drug exposure, channels were washed with 3 × 100 μL DMEM and then incubated for a further 24 h before staining and analysis.

### 2.8 Ultrasound Instrumentation and Exposure

Microfluidic channels were exposed to the US using an unfocused, 2.25 MHz central frequency transducer (V323-SM, Olympus, US) with an element diameter of 6.35 mm. A computer-controlled function generator (TG5011A, Aim-TTi, UK) generated US pulses, which provided sinusoidal burst cycles to a +53 dB power amplifier (A150, E&I Ltd., USA). Each US exposure used the following parameters: mechanical index, 0.6, driving frequency, 2.25 MHz, peak negative pressure, 900 kPa, pulse repetition frequency, 1 kHz, duty cycle, 1%, and total duration 5 s. A 3D-printed housing was used to hold the microfluidic chip and transducer. A 20 mm stand-off pad and coupling gel were used to ensure consistent contact and distance between the transducer and microfluidic channels and to maintain far-field US conditions. The chip housing was placed across a water bath containing an acoustic absorber to minimize reflections and the formation of standing waves.

### 2.9 Live/Dead Cell Staining and Confocal Imaging

24 h post-treatment, cells were washed with 3 × 100 μL serum-free DMEM, then stained with 3 × 100 μL of a solution of 2 μM Calcein-AM and 4 μM Ethidium Homodimer III in serum-free DMEM. Cells were incubated with staining solution for 30 min and then washed with 3 × 100 μL serum-free DMEM before imaging using confocal laser scanning microscopy (CLSM). A Leica DMi8/SP8 confocal microscope was used for fluorescent imaging of cells treated in the microfluidic channels. Calcein-AM and Ethidium Homodimer III were excited sequentially using 488 and 552 nm OPSL diode lasers, respectively. Fluorescence emission for Calcein-AM was measured from 493 to 586 nm and for Ethidium Homodimer III between 590 and 749 nm. A 10× objective was used, and the confocal pinhole was set to 2 a.u., giving an imaging depth of 19 μm, allowing us to image only cells adhered to the top face of the channel. Full images of each channel were generated using the TileScan feature, which captures multiple 512 × 512 pixel images and then merges, creating a complete image of the channel. A 9-point focus map was used to obtain the TileScan images, and images were analyzed with a custom MATLAB (R2020a, MathWorks, USA) script. Using LS174T (13.6 μm)^40^ and HCT116 (18.4 μm)^41^ diameters from the literature in conjunction with our imaging scale, it is calculated that a typical cell consists of 28–52 pixels.

## 3 Results and Discussion

### 3.1 LS174T Cell Response to Free AA and PAL

To determine the short exposure cytotoxicity of AA and PAL, LS174T cells grown in 96-well plates were exposed to a range of AA and PAL concentrations for 1 h (see methods Sections 2.3 and 2.5, and Supporting Information Figure S1). AA exhibited a dose-dependent cell killing effect, with an IC_50_ value of 3.6 mM (Supporting Information Figure S2), in agreement with the literature for LS174T, HT-29, and SW480 CRC cells.^42^ This provides a target concentration for the transition to encapsulated delivery. Due to the potential detrimental side effects^16^ and instability^15,29^ of free vitamin C, its PA variant was intercalated into liposomes at 30 mol % following a minor variation of the method of Sawant et al.^30^ PAL showed minimal batch-to-batch variation, with a typical batch (before concentrating by centrifugation) exhibiting an average size of 126 ± 4 nm and a concentration of 3.4 ± 0.2 × 10^13^ particles/mL (*n* = 3 ± SE), see Supporting Information Figure S3. The PA concentration present in PAL samples used for drug treatments was determined to be ∼100% by HPLC, as expected, due to the hydrophobic nature of the PA and previously reported literature values for PA incorporation into liposomes.^28−30^

**Figure 1:**
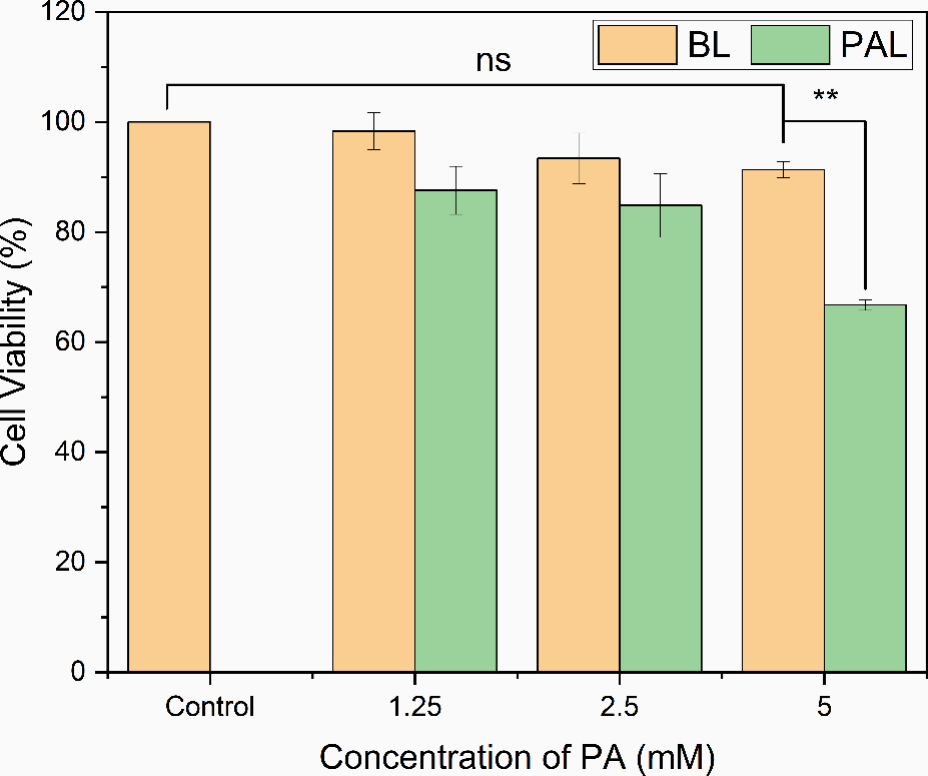
LS174TIn VitroCell Response to PAL. LS174T cell viability following 1 h exposure to PAL and BL. The bar chart shows treatment with PAL compared to the carrier (BL), which used the same liposome preparation method and yielded a similar liposome concentration but omitted PA (*n* = 3, error bars represent standard error for three biological repeats).

Figure 1, shows that PAL can be delivered to LS174T cells at cytotoxic concentrations while administered in a nonharmful vehicle (control, PBS + DMEM). Of the loadings considered, 5 mM PAL was the only condition to show a statistically significant reduction in cell viability compared to the control (****p < 0.0001). 5 mM PAL also showed a statistically significant (**p = 0.0022) reduction in remaining viable cells in comparison to a corresponding BL formulation, with 67 and 91% of cells remaining viable, respectively. BL were prepared using the same liposome preparation method and yielded a similar liposome concentration to PAL, but omitted PA, to discern any toxicity from the liposomal vehicle.

Cell viability decreased in a dose-dependent manner as PAL concentration increased, demonstrating that ascorbate retains its cytotoxicity when incorporated into liposomes. Notably, the BL were shown to be non-harmful to cells, indicating that including PA generates the anti-cancer effect.

Our findings align well with previous studies pertaining to PAL treatment of other cancer cell lines. With 1.6–7.5 mM PAL providing dose-dependent cytotoxicity against human breast tumor cell lines MCF7 and BT20, ovarian carcinoma A2780, renal adenocarcinoma ACHN, and mouse renal cancer RAG cells,^15^ while Sawant et al.^30^ reported cytotoxicity in 4T1 (murine) and MCF7 (human) cells using 3.75 mM PAL. Our 5 mM PAL condition, while showing modest cytotoxicity, was chosen to allow the study of potential synergistic effects when combined with MB and US.

### 3.2 Enhancing LS174T Cell Response to PAL with MBs + US

**Figure 2:**
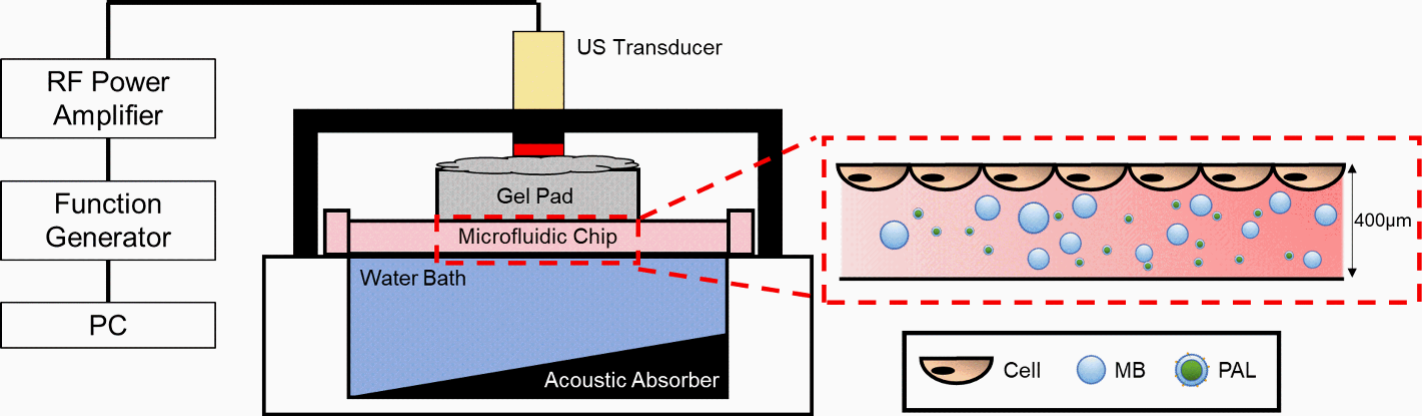
Schematic of the set-up used to treat cells with PAL, MBs, and US. The 6.35 mm diameter US transducer (mechanical index, 0.6, driving frequency, 2.25 MHz) was coupled to the microfluidic chip using acoustic gel and a gel pad. The zoomed region shows a representation of cells cultured on the top face of the microfluidic device and the PAL/MB formulation in the channel (not to scale). Adapted from Batchelor et al.,^38^ with permission under a CC BY 4.0 license. Copyright 2022 Langmuir.

MB and US enhancement of the PAL treatment was explored using the co-delivery method, with the experimental setup outlined in Figure 2. Cells were cultured on the top surface of microfluidic channels (Ibidi chips), such that the rising of MBs (10 min rise time, informed by models of the Hadamard-Rybczynski equation^39^), due to their buoyancy, brought them in contact with the cells. MBs were produced using the Horizon microfluidic platform^36^ and characterized using bright-field microscopy and a custom MATLAB script.^37^ MB characterization is given in Supporting Information Figure S4. Previous studies by our group showed that after diluting a concentrated stock of Horizon produced MBs (same gas and shell composition) to a concentration of 10^8^ MBs/mL, the MBs demonstrated an unchanging population after ∼1 h incubation in cell culture media at 37 °C.^43^ This confirms that on the time scales of our multi-exposure experiments, the MBs are present at the desired concentrations for each exposure.

**Figure 3:**
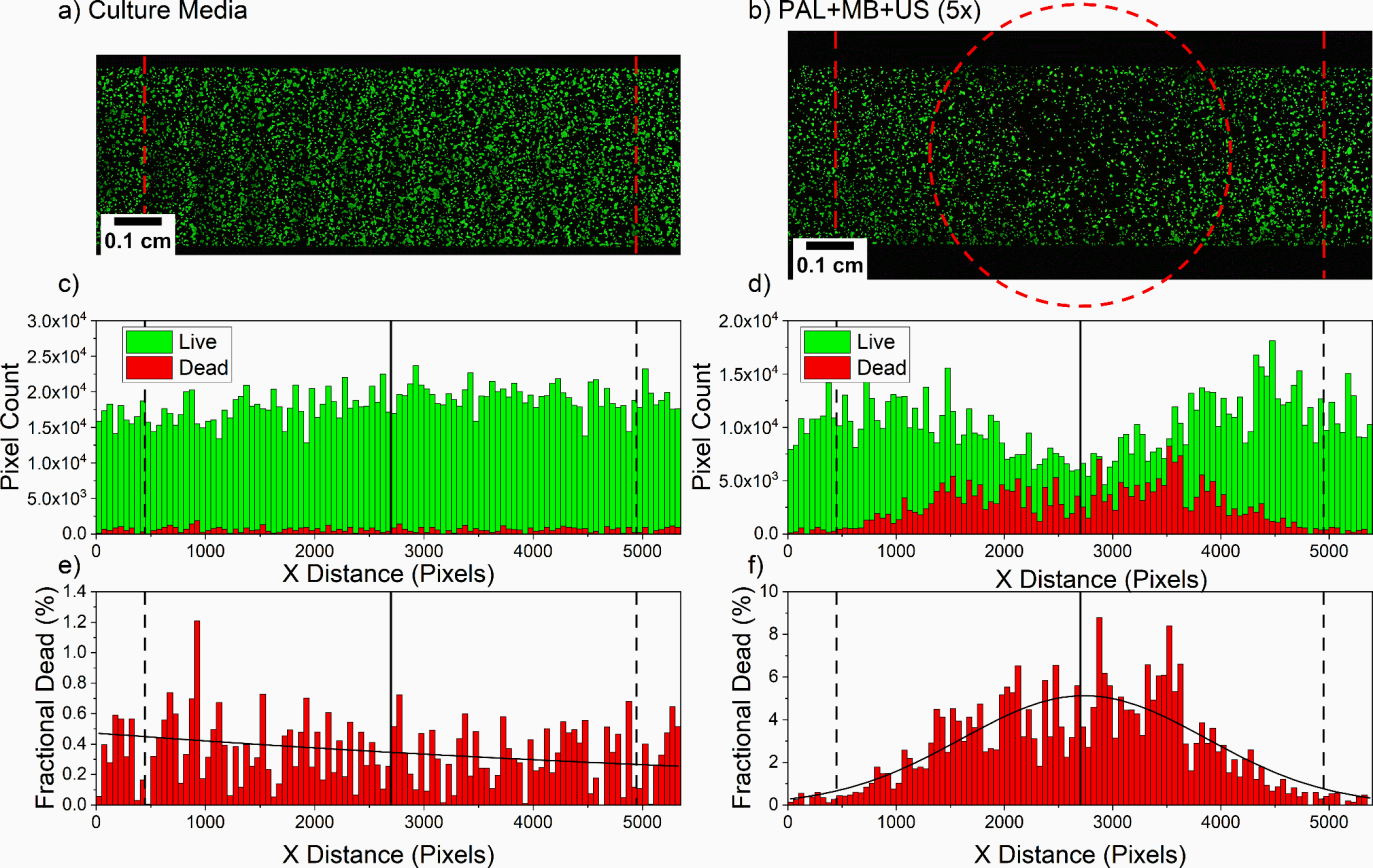
Fluorescence maps and analysis of LS174T cells cultured on-chip and treated with PAL, MBs, and US. Treatment with (a) culture media only, or (b) PAL + MB + US (5×). Histograms showing the distribution of live and dead stained cells (dead pixel count multiplied by 10 to aid visualization), corresponding to the confocal images of LS174T cells post-treatment with (c) culture media or (d) PAL + MB + US (5×). Histograms showing the distribution of the fraction of dead LS174T cells (dead pixel count/total pixel count) which received treatment with (e) culture media only or (f) PAL + MB + US (5×).

For treatment, the LS174T cells were exposed to various PAL, MB, and US combinations, with the treatment schedule outlined in Section 2.7 and Supporting Information Figure S5a, with a detailed timeline of the multiple exposure treatments provided in Supporting Information Figure S5b. To assess cell viability, cells were stained using Calcein-AM (stains live only) and Ethidium Homodimer III (stains dead only) and imaged using CLSM. Fluorescence maps were generated over the entire channel area for each treatment formulation, and an example is given in Figure 3a, with additional maps in Supporting Information Figure S6.

Image analysis was conducted with MATLAB and used the assumption that cells damaged by the treatment become detached from the top face of the microfluidic chip and wash away; this is supported by a control in which a 10% (v/v) ethanol treatment led to no remaining cells (live or dead). Hence, by calculating the total live (green) fluorescence of a treated channel and comparing it against the total live (green) fluorescence of a culture media (DMEM) only channel, the reduction of live cells was estimated.

Figure 3a and Figure 3b show confocal maps of channels that either received no treatment or were treated with PAL + MB + US (5×), respectively. The corresponding histograms shown in Figure 3c and Figure 3d were generated by vertical summations of the live (green) and dead (red) pixels in the images, with the dead pixel count multiplied by 10 to aid visualization. The PAL + MB + US (5×) treatment shows a reduction in the live cells and an increase in the dead cell intensity in the center of the channel, where the US was targeted (red dashed circle, Figure 3b). This demonstrates the treatment is localized to the area in which the US is delivered, this is vital in the reduction of detrimental systemic chemotherapy side effects^4^ and is a key objective of targeted drug delivery systems. Figure 3e and Figure 3f show the fractional dead percentage (dead pixel count/total pixel count) as calculated for each bin of the corresponding histograms presented in Figure 3c and Figure 3d. A Gaussian fit was applied to the PAL + MB + US (5×) condition (Figure 3f), further confirming the region of highest cell death was localized to the center of the transducer region, with greatly reduced cell death observed outside of the transducer region. Fluorescence maps and corresponding histograms for other conditions trialled are summarized in Supporting Information Figure S6. In all cases, the dead pixel count has been multiplied by 10 to aid visualization. The fluorescence map of the PAL + MB + US (1×) (Supporting Information Figure S6a) showed no significant reduction in live cells. The decision to trial the addition of fresh PAL + MB formulations and further US applications was inspired by a recent study showing improved anti-cancer efficacy when multiple treatments with MBs and US were used.^44^ The localized cell killing effect was also observed in the PAL + MB + US (3×) (Supporting Information Figure S6b), MB + US(5×) (Supporting Information Figure S6c), and BL + MB + US (5×) (Supporting Information Figure S6d) cases.

**Figure 4:**
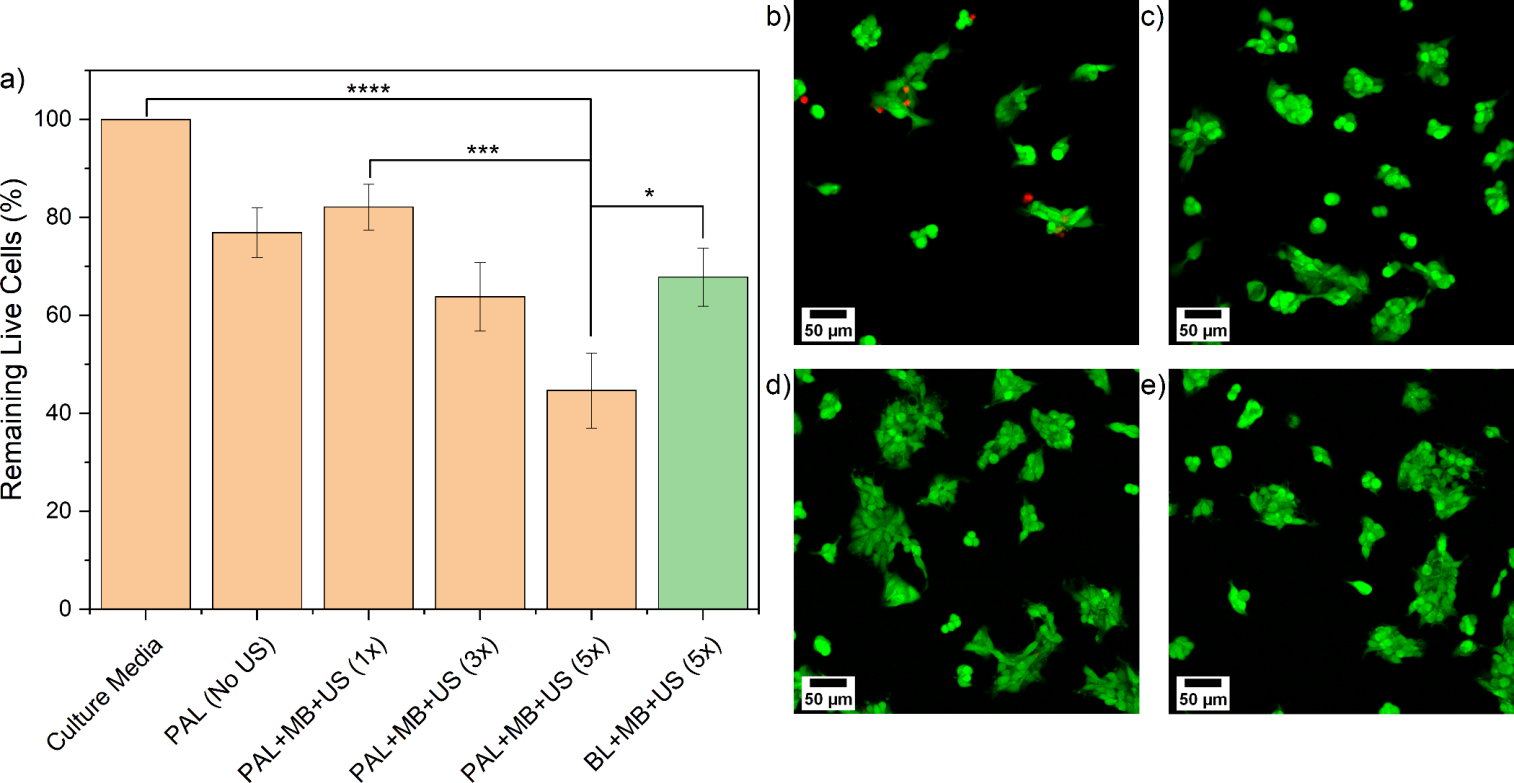
LS174T cell viability post BL, PAL, MB, and US exposures on-chip. (a) Cell viability data for LS174T cells on-chip, post-treatment with BL, PAL (5 mM), MB (1 × 10^8^ MBs/mL), and US combinations (*n* > 3 for all conditions, except for PAL (No US), conducted twice; error bars represent the standard error). Confocal images of stained LS174T cells following treatment with PAL + MB + US (5×) (b) inside and (c) outside of the US-exposed region. Confocal images of stained LS174T cells treated with culture media (DMEM) in the channel’s (d) central and (e) edge regions.

The total live (green) pixel count within a fixed region (between the red and black dashed vertical lines, Figure 3) was calculated for each image, and this value was compared to the total live (green) pixel count of the untreated channel, to calculate the percentage reduction in live cells. The reduction in live cells was used as our metric to indicate the efficacy of each treatment and data for the LS174T cells is presented in Figure 4. The control consisted of PBS, PBS + 1% glycerol, and DMEM at the same concentration used to deliver PAL (in PBS) and MB (in PBS + 1% glycerol), ensuring any observed cytotoxicity was not caused by the solvent/vehicle. DMEM and control showed no significant difference, as shown in Supporting Information Figure S7. PAL delivered without MBs or US (PAL (No US)) compared to PAL delivered with MBs and US (PAL + MB + US (1×)) showed no minimal difference, leaving 77 and 82% of cells remaining compared to DMEM, respectively. However, it was found that introducing a fresh formulation, allowing a 10 min MB rise time, then re-applying the US, an increased cytotoxic effect was realized. For instance, PAL + MB + US (3×) and PAL + MB + US (5×) saw the percentage of viable cells remaining reduced to 64% (****p < 0.0001) and 45% (****p < 0.0001) respectively when compared to DMEM. Close-up confocal images of cells treated with PAL + MB + US (5×) show more dead cells and a lack of remaining live cells in the central, US-exposed region of the microfluidic channels (Figure 4b) in comparison with areas toward the edge of the channel, non-US exposed (Figure 4c) and no observable difference across the DMEM only channel (Figure 4d and Figure 4e). Crucially, the gradual improvement in therapeutic outcome observed as we move from PAL (No US) to PAL + MB + US (1×), (3×) and (5×) was achieved without increasing the drug dosage, or exposure time. The incremental effect of multiple exposures to MB and US demonstrated here with PAL could be used to maximize the therapeutic outcome for a range of chemotherapeutic treatments.

After MB + US (5×) treatment, the percentage of remaining viable cells was 72% (**p = 0.0017) compared to DMEM (Supporting Information Figure S7). BL + MB + US (5×) was tested and 68% (***p = 0.0003) of cells remained viable compared to DMEM. The comparison of PAL to BL treatments shows the use of PAL provided a further 23% reduction in viable cells, indicating the importance of the presence of PA in obtaining the cytotoxic effect (*p = 0.0347 when comparing BL + MB + US (5×) and PAL + MB + US (5×)). PAL + MB + US (5×) was shown to offer a reduction in cell viability when compared to PAL (No US), MB + US (5×) (**p = 0.0074) and PAL + MB + US (1×) (***p = 0.0002). Additional control experiments are presented in Supporting Information Figure S7, showing that vehicle control, US alone (5×) and exposure to BL (no US) did not affect LS174T cell viability.

### 3.3 Enhancing HCT116 Cell Response to PAL with MBs + US

**Figure 5:**
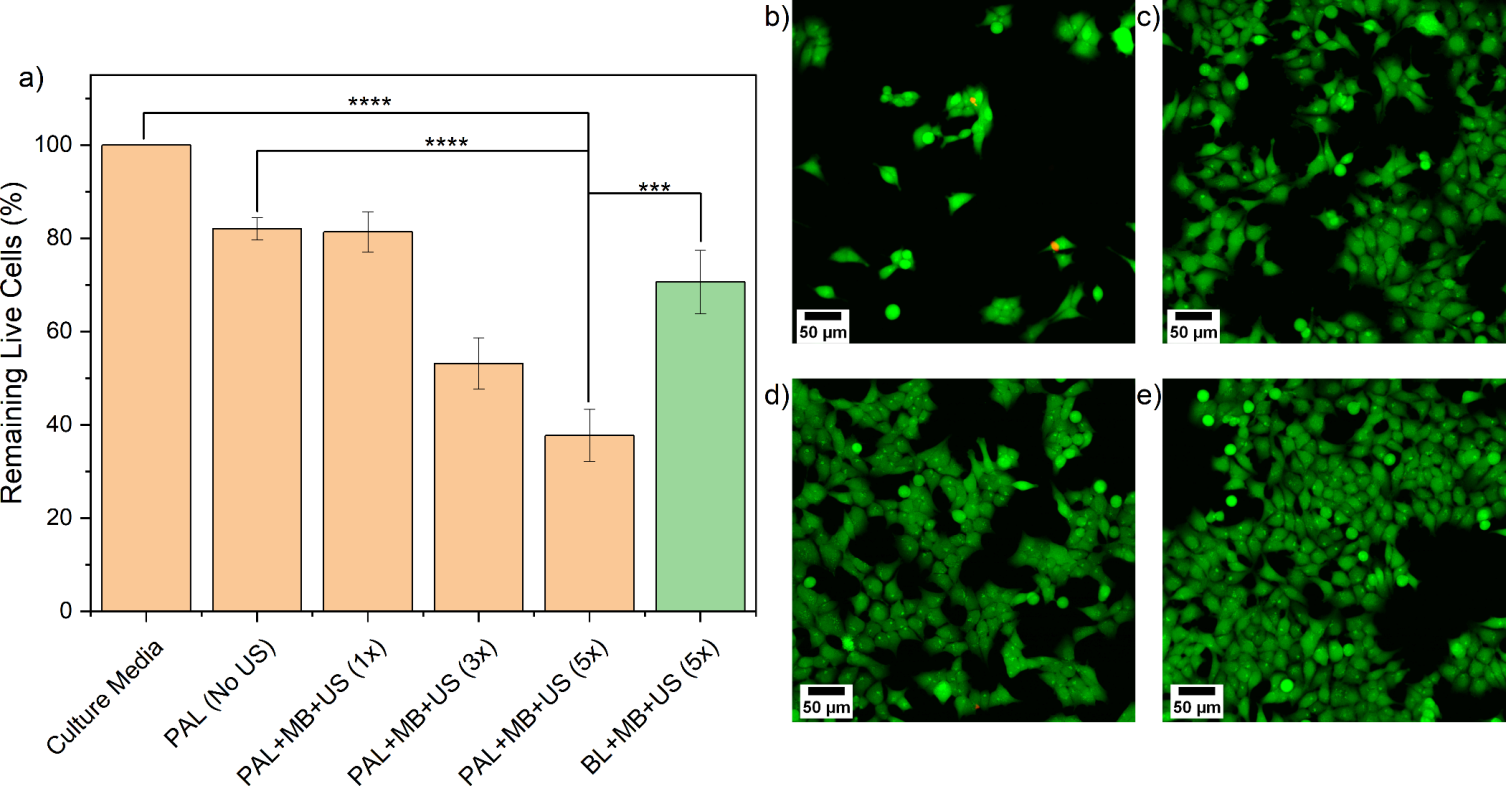
HCT116 cell viability post BL, PAL, MB, and US exposures on-chip. (a) Cell viability data for HCT116 cells on-chip, post-treatment with BL, PAL (5 mM), MB (1 × 10^8^ MBs/mL), and US combinations (*n* > 3 for all conditions, and error bars represent standard error). Confocal images of stained HCT116 cells following treatment with PAL + MB + US (5×) (b) inside and (c) outside of the US-exposed region. Confocal images of stained HCT116 cells treated with culture media (DMEM) in the channel’s (d) central and (e) edge regions.

To ensure the observed effects were not restricted to the LS174T cells, the experiment was repeated using HCT116 cells (see Supporting Information Figure S8), which is also a KRAS-mutated CRC cell line. The key findings are summarized in Figure 5, and a detailed analysis is provided in Supporting Information Figure S9. The PAL + MB + US (5×) treatment showed a significant reduction (38% viable, Figure 5a) of the live cell intensity, in the center of the treatment zone. The localized cell killing effect was also observed in the PAL + MB + US (3×) (53% viable, Supporting Information Figure S9c), MB + US(5×) (66% viable, Supporting Information Figure S9f), and BL + MB + US (5×) (71% viable, Supporting Information Figure S9h) treatments. After the treatment of HCT116 cells with PAL + MB + US (3×) (Figure S9c), fewer dead pixels were observed than in the PAL + MB + US (5×) case (Figure S8d). This is likely caused by the PAL + MB + US (3×) treatment damaging the cells, but not removing them from the chip. However, as PAL + MB + US (5×) leads to additional damage, this causes the dead cells to detach from the chip and then removed by subsequent wash steps.

Analogous to the LS174T results, higher magnification images of cells treated with PAL + MB + US (5×) the removal of live cells in the central, US exposed region of the microfluidic channels (Figure 5b) in comparison with areas toward the edge of the channel, non-US exposed (Figure 5c) and no observable difference across the DMEM only channel (Figure 5d and Figure 5e). Additional control experiments were conducted and are presented with detailed analysis in Supporting Information Figure S10.

## 4 Conclusions

This study investigated the therapeutic effect of PAL toward KRAS-mutated CRC cell lines and the use of MB and US to enhance localized delivery to tumor cells. Due to the poor stability of AA in the bloodstream, the acyl chain linked analog PA was used. PA inserts into hydrophobic carriers, such as liposome shells, protecting the ascorbate moiety. It was shown that PAL generated a cytotoxic effect against LS174T cells in a well plate system. Subsequent on-chip experiments observed that using US to induce MB cavitation enhanced the efficacy of PAL treatment ∼2-fold in both LS174T and HCT116 CRC cell lines. Fluorescence image analysis demonstrated that the therapeutic effect was localized to the area that received the US pulse and was enhanced in the case of PAL + MB + US compared to MB + US or PAL alone; this is critical for developing delivery systems that minimize off-site toxicity. It was also shown that multiple exposures to MBs and US raised the therapeutic effect compared to using a single exposure.

## Supporting Information Available

Timeline of well plate treatments; LS174T in vitro cell response to AA; Characterization of PAL; MB size distribution; Timeline of on-chip treatments; Additional confocal fluorescence maps for LS174T cells post-treatment on-chip; LS174T cell viability post BL, PAL, MB and US control exposures on-chip; Fluorescence maps and analysis of HCT116 cells cultured on-chip and treated with PAL, MBs and US; Additional confocal fluorescence maps for HCT116 cells post-treatment on-chip; HCT116 cell viability post BL, PAL, MB and US control exposures on-chip. Data used in the figures of this paper are openly available from the University of Leeds data repository 10.5518/1339 (PDF)

## Abbreviations

CRC: colorectal cancer
AA: ascorbic acid
GLUT-1: glucose transporter 1
MB: microbubble
US: ultrasound
PA: palmitoyl ascorbate
PAL: palmitoyl ascorbate liposomes
DMEM: Dulbecco’s modified eagle medium
DPBS: Dulbecco’s phosphate-buffered saline
DPPC: 1,2-dipalmitoyl-*sn*-glycero-3-phosphocholine
DSPE-PEG2000: 1,2-distearoyl-*sn*-glycero-3-phosphoethanolamine-N-[methoxy(polyethylene glycol)-2000]
PBS: phosphate-buffered saline
ANOVA: analysis of variance
BL: blank liposomes
UV–vis-NIR: ultraviolet–visible-near-infrared spectroscopy
DAD: diode array detector
HPLC: high performance liquid chromatography
CLSM: confocal laser scanning microscopy
