# Supplementary material for: Microbubble Enhanced Delivery of Vitamin C for Treatment of Colorectal Cancer
Source: ACS Omega. 2024 Oct 30;9(45):45270–8. doi: 10.1021/acsomega.4c06779 (PMC11561761; doi:10.1021/acsomega.4c06779)
Supplement: Supplementary file 1 — ao4c06779_si_001.pdf [file ao4c06779_si_001.pdf]

Supporting Information

Microbubble Enhanced Delivery of Vitamin C for Treatment of Colorectal Cancer

Joseph Fox[1], Damien V. B. Batchelor[1], Patricia Louise Coletta[2], Elizabeth M.A. Valleley[2] and Stephen D. Evans[1].*

[1]Molecular and Nanoscale Physics Group, School of Physics and Astronomy, University of Leeds, LS2 9JT, United Kingdom

[2]Leeds Institute of Medical Research, Wellcome Trust Brenner Building, St James's University Hospital, Leeds, LS9 7TF, United Kingdom

*Corresponding author E-mail: s.d.evans@leeds.ac.uk

1 Supporting Information

1.1 Well Plate Treatment Schedule

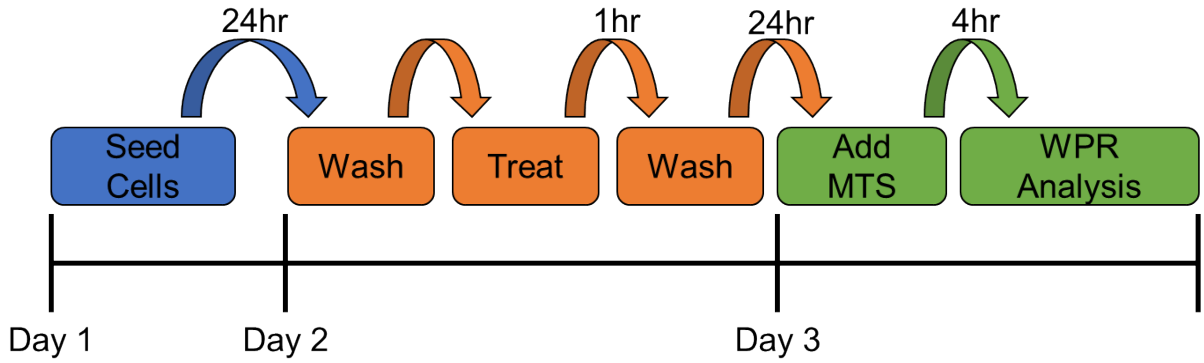

Figure S1 Timeline of well plate treatments. Cells were seeded into 96-well plates on day 1 and left to adhere for 24 h. On day 2, cells were washed, treated with drug formulation for 1 h, washed, then incubated for a further 24 h. On day 3, MTS reagent was added to the treated wells, incubated for 4 h, then analyzed with the well plate reader.

1.2 LS174T *In Vitro* Cell Viability Analysis After Exposure to AA and PAL

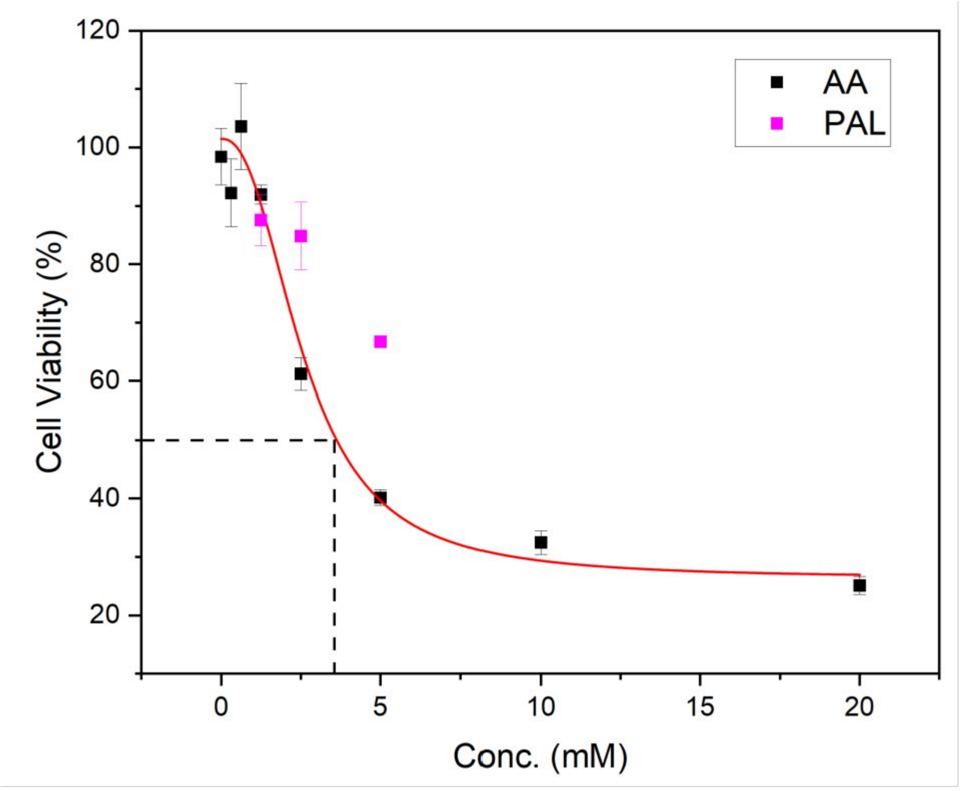

Figure S2 LS174T *In Vitro* Cell Response to AA. LS174T cell response following 1 h exposure to AA (n=3, error bars represent standard error for 3 biological repeats, dashed line shows IC_{50}). LS174T cell response following 1 h exposure to PAL included for ease of comparison.

1.3 Liposome Characterisation

a)

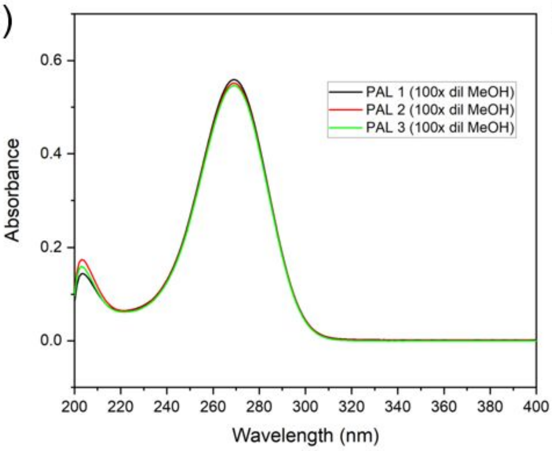

b)

Sample	Liposome Conc. (x10^{13} Particles/mL)	Liposome Mean Diameter (nm)
**PAL 1**	3.75 ± 0.2	119 ± 1
**PAL 2**	2.95 ± 0.2	128 ± 2
**PAL 3**	3.37 ± 0.1	131 ± 3
**BL 1**	2.43 ± 0.1	132 ± 2
**BL 2**	1.93 ± 0.1	146 ± 4
**BL 3**	1.7 ± 0.09	132 ± 3

Figure S3 Characterization of PAL. a) UV-Vis spectra of PAL diluted in methanol. Samples show minimal spectral variation batch to batch. b) Nanoparticle Tracking Analysis data for 3 samples of PAL and 3 samples of BL. Samples show minimal variation in concentration and size batch to batch.

1.4 Microbubble Characterisation

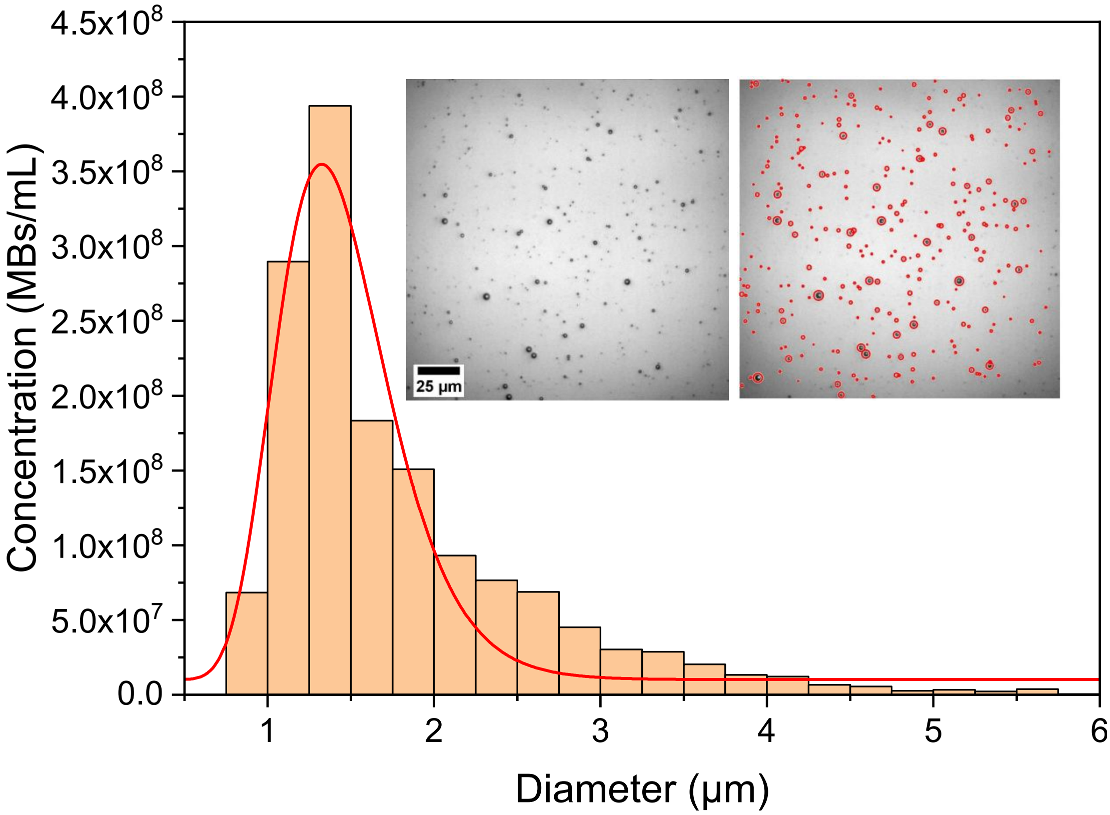

Figure S4 Representative MB population. Plot showing size distribution of a typical sample of MBs produced using the microfluidic platform. Insets show a typical bright-field image along with the image post circle fitting for analysis. For this sample, concentration = $1.5 \times 10^9 \pm 1.45 \times 10^8$ MBs/mL, mean diameter = 1.8 ± 1 μm and number of MBs analyzed = 2725.

1.5 On-Chip Treatment Schedule

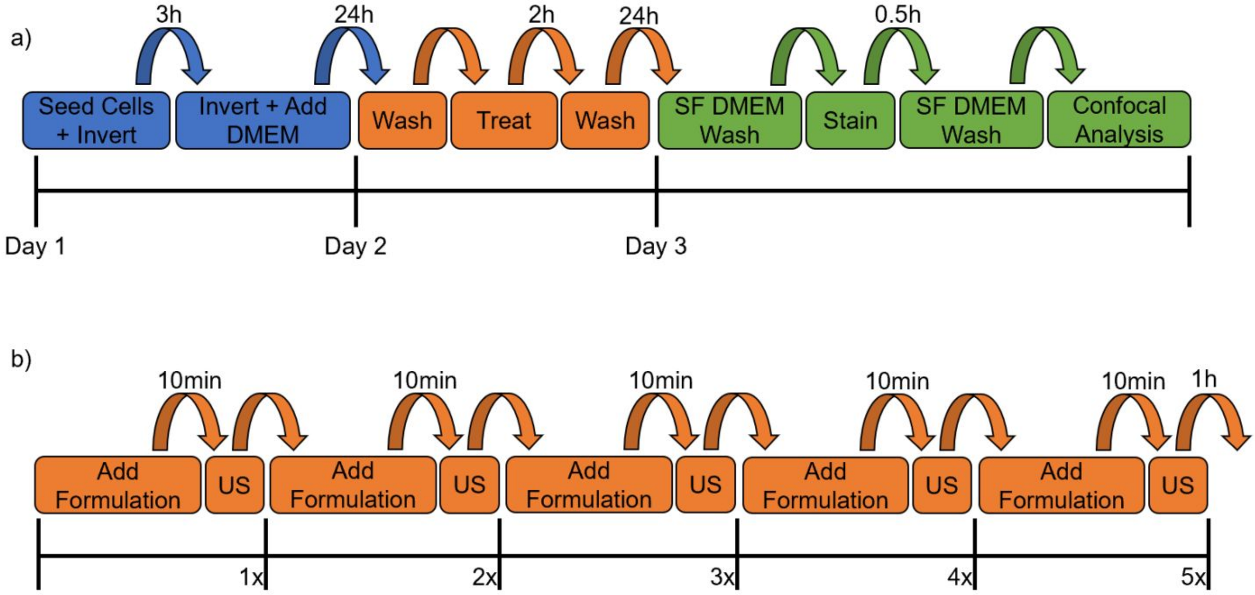

Figure S5 Timeline of on-chip treatments. a) Day 1, cells were seeded into the channels of the Ibidi chip and quickly inverted before incubation for 3 h. After 3 h, chips were inverted again, such that they were the correct way up and DMEM was added to both reservoirs of each channel simultaneously, before returning chips to the incubator for 24 h. On day 2, channels were washed, exposed to treatment formulation for 2 h, washed, then returned to the incubator for 24 h. On day 3, channels were washed with serum-free media, incubated with stain for 30 min, washed with serum-free media and then analyzed by confocal microscopy. b) Expanded timeline of the treatment schedule when multiple MB and US exposures used. For all treatments, the total duration of formulation exposure was the same, hence, if a channel was only receiving 1x US exposure, that channel remained exposed to the drug whilst the remaining exposures were conducted for the channels receiving multiple exposures.

1.6 LS174T Additional Post-Treatment Fluorescence Maps

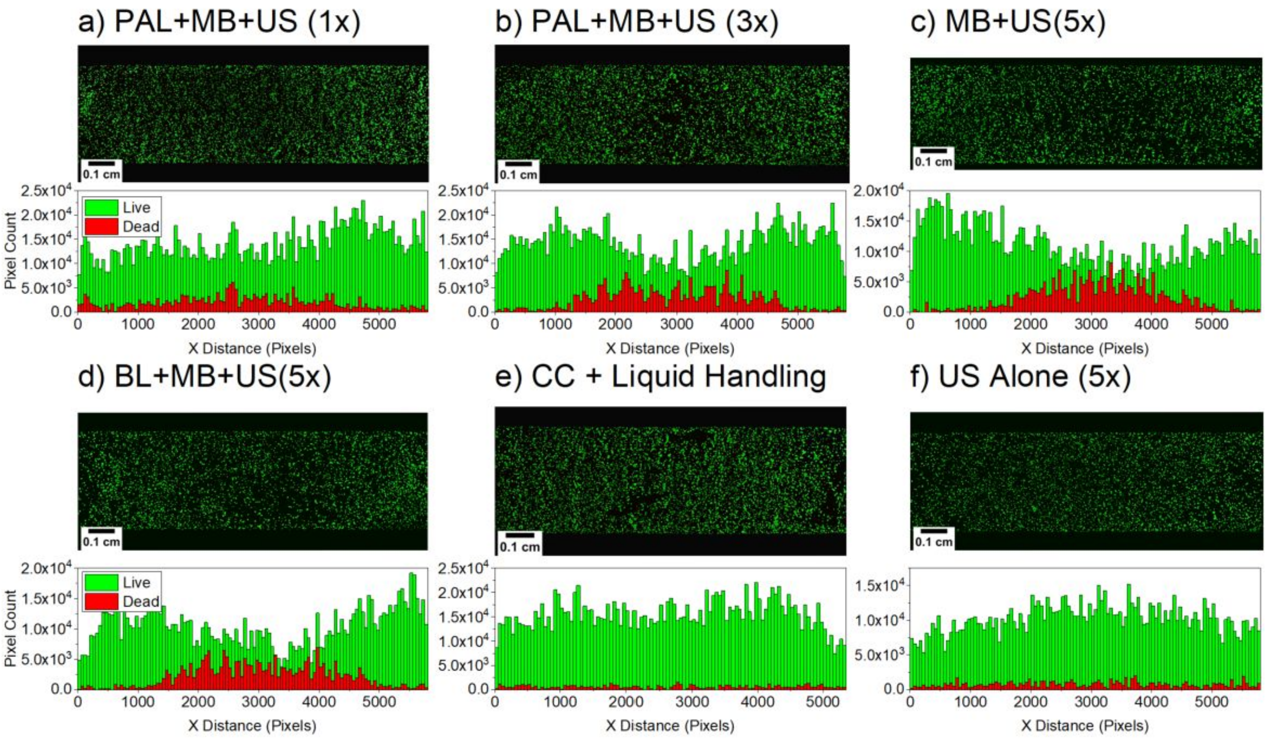

Figure S6 Additional confocal fluorescence maps for LS174T cells post-treatment on-chip. Confocal fluorescence maps and histograms showing distribution of live and dead stained pixels, corresponding to the confocal images of LS174T cells which received treatment with a) PAL+MB+US (1x), b) PAL+MB+US (3x), c) MB+US (5x), d) BL+MB+US (5x), carrier control with liquid handling (CC + Liquid Handling) and f) US Alone (5x). Dead pixel count multiplied by 10 in all cases, to aid visualization.

1.7 Enhancing LS174T Cell Response to PAL with MBs + US (Controls)

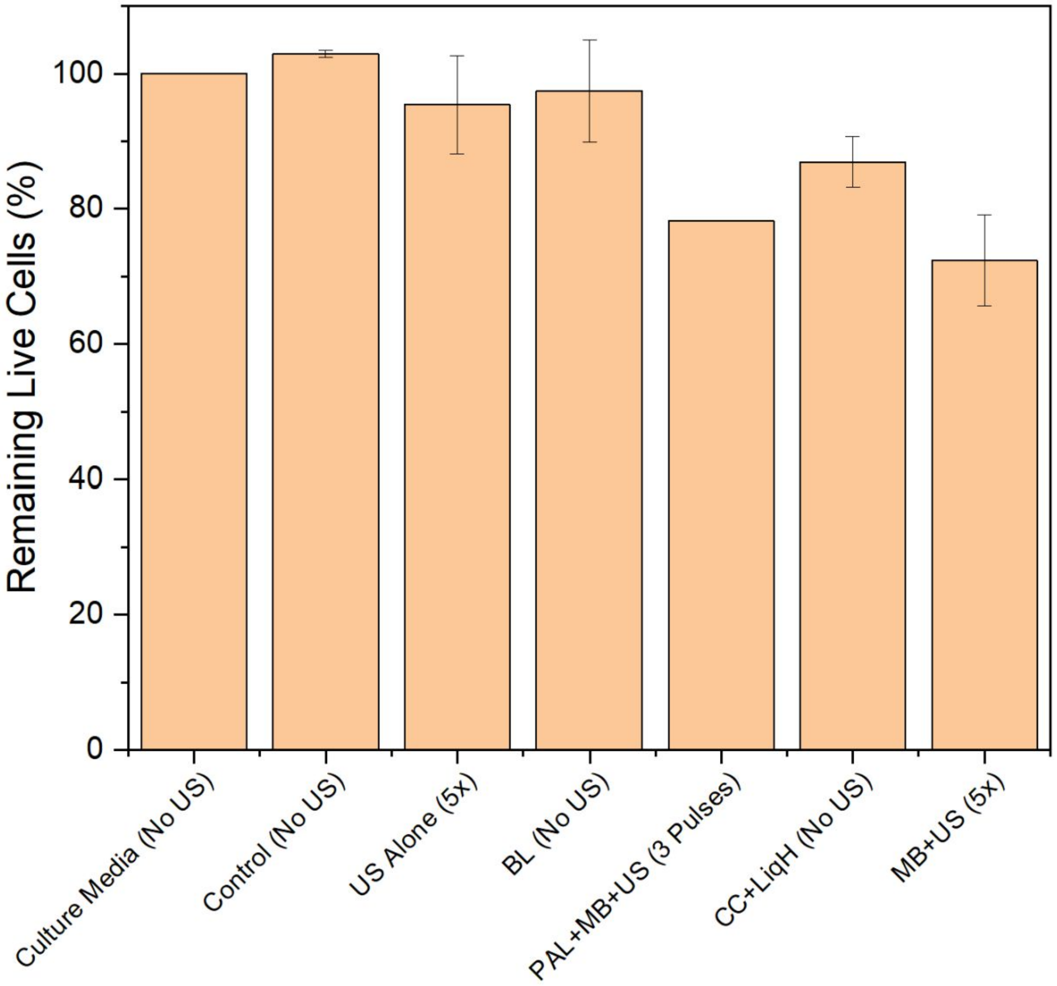

Figure S7 LS174T cell viability post BL, PAL, MB and US control exposures on-chip. Each data point shows mean ± standard error and consists of over 3 repeats, except for US Alone (5x) and BL (No US), conducted twice.

1.8 Fluorescence maps and analysis of HCT116 cells

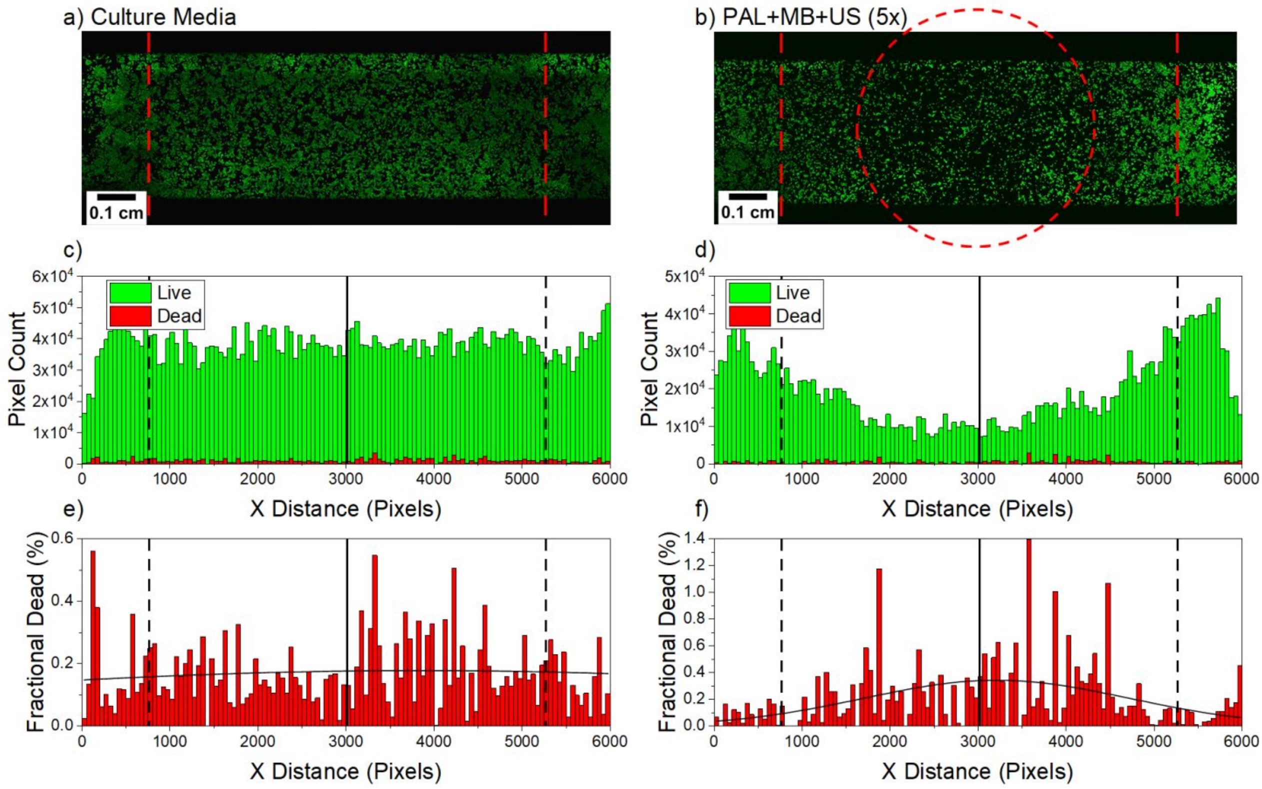

Figure S8 Fluorescence maps and analysis of HCT116 cells cultured on-chip and treated with PAL, MBs and US. Confocal fluorescence maps of HCT116 cells which received treatment with a) culture media only, or b) PAL+MB+US (5x). Histograms showing distribution of live and dead stained pixels (dead pixel count multiplied by 15 to aid visualization), corresponding to the confocal images of LS174T cells which received treatment with c) culture media only, or d) PAL+MB+US (5x). Histograms showing distribution of the fraction of dead cells LS174T cells, which received treatment with e) culture media only, or f) PAL+MB+US (5x), with Gaussian fit applied.

1.9 HCT116 Additional Post-Treatment Fluorescence Maps

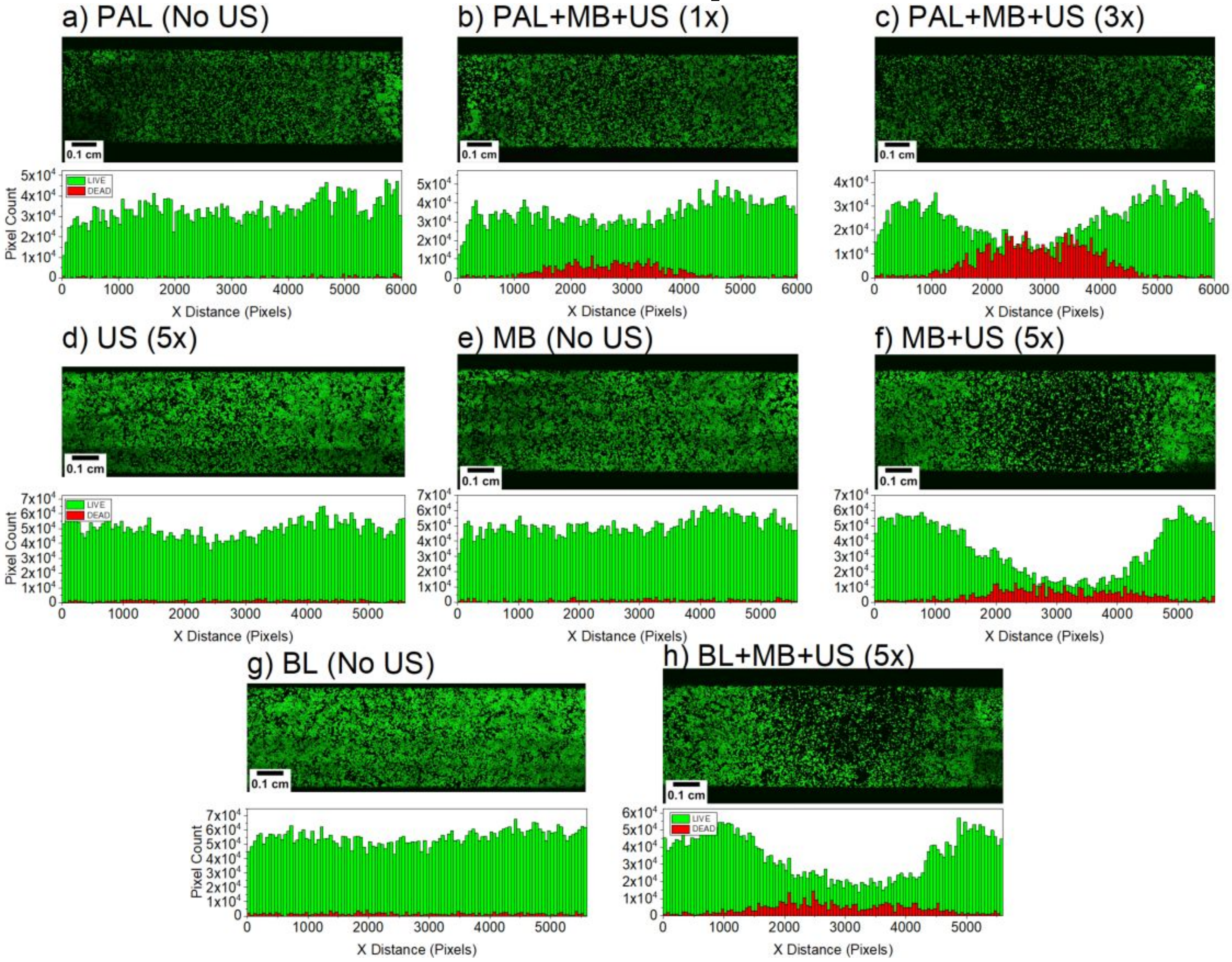

Figure S9 Additional confocal fluorescence maps for HCT116 cells post-treatment on-chip. Confocal fluorescence maps and histograms showing distribution of live and dead stained pixels, corresponding to the confocal images of HCT116 cells which received treatment with a) PAL (No US), b) PAL+MB+US (1x), c) PAL+MB+US (3x), d) US (5x), e) MB (No US), f) MB+US (5x), g) BL (No US) and h) BL+MB+US (5x). Dead pixel count multiplied by 15 in all cases, to aid visualization.

PAL delivered without MBs or US (PAL (No US)) compared to PAL delivered with MBs and US (PAL + MB + US (1x)) showed no significant difference between each other, leaving

82% and 81% of cells remaining compared to DMEM, respectively. However, it was found that by introducing a fresh formulation, allowing 10 min MB rise time, then re-applying US, an increased cytotoxic effect was realized. For instance, PAL+MB+US (3x) and PAL+MB+US (5x) saw the percentage of viable cells remaining reduced to 53% (****$p<0.0001$) and 38% (****$p<0.0001$) respectively when compared to DMEM.

Post-treatment with BL+MB+US (5x), 71% of cells remained viable, giving a statistically significant difference compared the PAL+MB+US (5x) condition (***$p=0.001$) and indicating the need for the inclusion of PA in the liposomal formulation to obtain the highest cytotoxic effect. PAL+MB+US (5x) showed a significant (****$p<0.0001$) reduction in cell viability when compared to PAL (No US), PAL+MB+US (1x), US Alone (5x), MB (No US) and MB+US (5x) (***$p=0.0006$).

1.10 Enhancing HCT116 Cell Response to PAL with MBs + US (Controls)

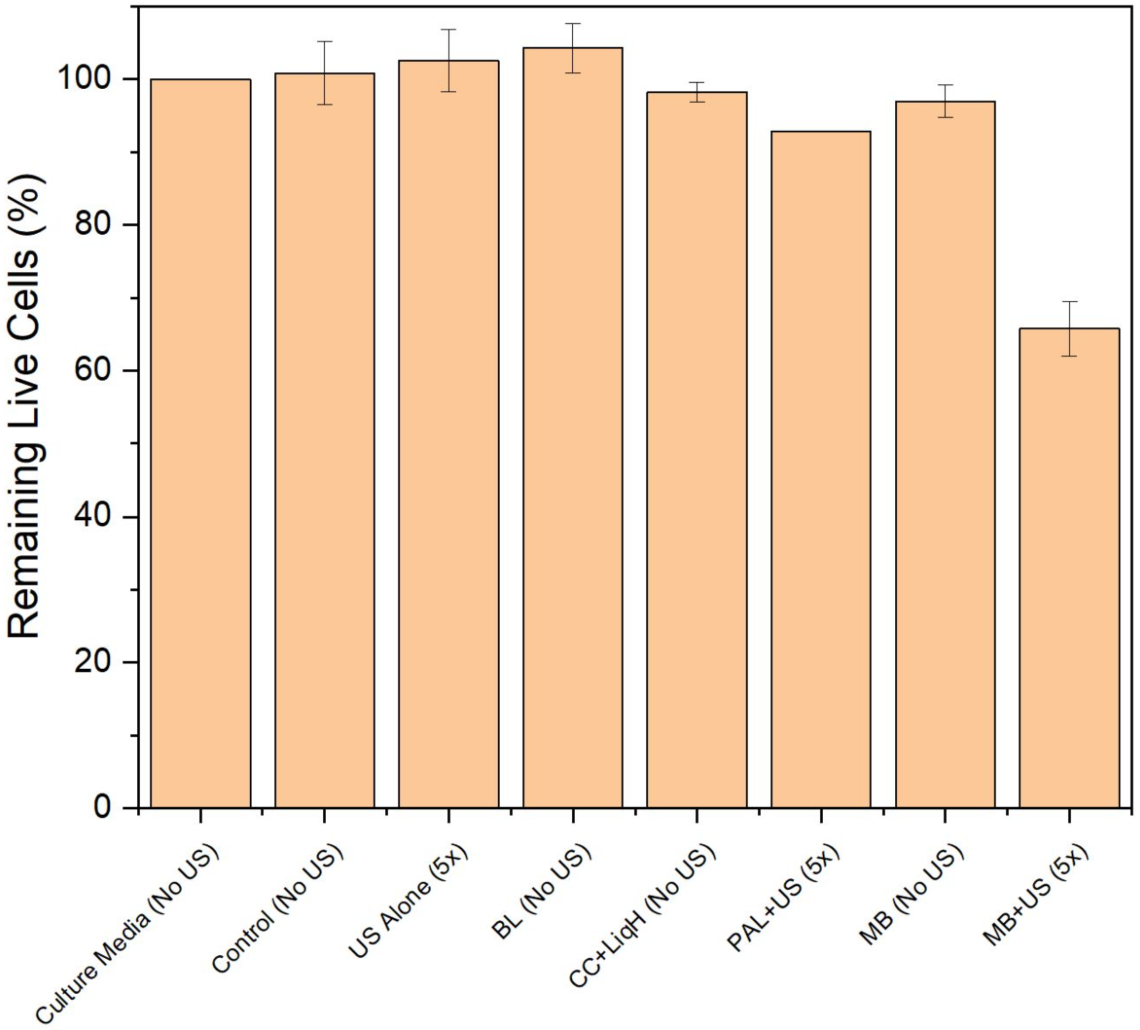

Figure S10 HCT116 cell viability post BL, PAL, MB and US control exposures on-chip. Each data point shows mean ± standard error and consists of over 3 repeats, except for PAL + US (5x), conducted once.

DMEM and control showed no significant difference. Control, US alone (5x) and BL (no US), control with liquid handling and MB (no US) were unharmful to HCT116 cells (viability > 95%, no statistical significance when compared to DMEM). The HCT116 cells appear to be more resistant to the liquid handling steps in comparison to LS174T. This is potentially due to the clumpy of nature of LS174T cells creating large aggregates which are more likely to be

disturbed by liquid exchanges, in comparison to a monolayer. Whilst MBs (no US) and US alone (5x) were unharmful to cells individually, when given in combination, MB+US (5x) showed a significant reduction in cell viability (66% viability remaining, ****$p<0.0001$) compared to DMEM only. This emphasizes that it is the interaction between the US and MBs that leads to cell death and shows the components to be unharmful when given alone. Again, regardless of the number of formulations added or formulation used, cells in all conditions were exposed to formulations for the same time.
